# PARK7 is a Key Regulator of Oxidative Stress - Related Breast Cancer Risk: A Multi-Omics Study

**DOI:** 10.7150/jca.111796

**Published:** 2025-06-23

**Authors:** Tianhua Wang, Yan Yao, Minpu Zhang, Hao Luan, Xinjie Chang, Lijuan Liu, Changgang Sun

**Affiliations:** 1 Faculty of Chinese Medicine and State Key Laboratory of Quality Research in Chinese Medicine, Macau University of Science and Technology, Macao.; 2 Department of Oncology, Weifang Hospital of Traditional Chinese Medicine, Weifang, China.; 3 Innovation Research Institute of Traditional Chinese Medicine, Shandong University of Traditional Chinese Medicine, Jinan, China.; 4 First Clinical Medical College, Shandong University of Traditional Chinese Medicine, Jinan, China.; 5 College of Traditional Chinese Medicine, Shandong Second Medical University, Weifang, China.

**Keywords:** Breast Cancer, Oxidative Stress, Multi-Omics, Mendelian Randomization, PARK7

## Abstract

**Background:** Oxidative stress (OS) is closely associated with the occurrence and progression of breast cancer (BC). However, its role as a potential etiological factor or trigger remains unclear. This study aims to systematically investigate the potential causal effects and mechanisms of OS-related genes in BC by integrating multi-omics data.

**Methods:** This study obtained summary data for blood methylation (mQTL), gene expression (eQTL), alternative splicing (sQTL), and protein abundance (pQTL) from their respective quantitative trait loci (QTL) studies. The genetic association data for breast cancer (BC) were primarily derived from the Breast Cancer Association Consortium (BCAC) and were validated using the UK Biobank (UKB) and FinnGen databases. SMR (Summary-data-based Mendelian Randomization) analysis was performed to evaluate the associations between the molecular characteristics of oxidative stress-related genes and BC. Subsequently, colocalization analysis was conducted to determine whether the identified signals share the same causal genetic variants. Whole-transcriptome association studies (TWAS), whole-proteome association studies (PWAS), and multi-marker analysis with genomic annotation (MAGMA) were used as sensitivity analyses. In addition, the significant genes were validated using multi-omics analysis of blood samples in an independent cohort from Weifang Traditional Chinese Medicine Hospital.

**Results:** By integrating multi-omics data from mQTL, eQTL, sQTL, and pQTL analyses, we identified PARK7 as a key oxidative stress-related gene that showed significant associations with breast cancer (BC) at multiple levels. Elevated gene expression of PARK7 (pSMR = 5.79E-06, OR = 0.91) and protein levels (pSMR = 8.46E-06, OR = 0.83) were significantly associated with reduced BC risk. In the mQTL analysis, cg10385390 (pSMR = 2.03E-03, OR = 1.09) and cg11518359 (pSMR = 3.54E-03, OR = 0.88) were significantly associated with BC. In the sQTL analysis, PARK7 (chr1:7961793-7962763) (pSMR = 8.87E-06, OR = 1.03) and PARK7 (chr1:7961735-7962763) (pSMR = 9.38E-06, OR = 0.97) were significantly associated with BC. In the integrated mQTL-eQTL SMR analysis, cg10385390 (pSMR = 7.61E-15, OR = 0.47) and cg11518359 (pSMR = 4.56E-09, OR = 2.78) exhibited significant associations. In the integrated sQTL-pQTL SMR analysis, PARK7 (chr1:7961793-7962763) (pSMR = 4.62E-44, OR = 0.85) and PARK7 (chr1:7961735-7962763) (pSMR = 6.56E-42, OR = 1.19) both showed significant associations. These findings revealed the multidimensional molecular mechanisms by which PARK7 regulates breast cancer risk through the oxidative stress pathway. All findings were supported by colocalization analysis (PPH4 > 0.7). Validation in an independent population cohort indicated that plasma levels of PARK7 mRNA and protein in breast cancer patients were significantly lower than those in healthy controls, consistent with the aforementioned results.

**Conclusion:** This study innovatively integrates multi-omics data to elucidate the complex associations between the PARK7 gene and breast cancer risk, providing new insights for precision prevention and targeted intervention of breast cancer.

## Introduction

The onset and progression of breast cancer (BC) are closely associated with the state of intracellular oxidative stress (OS), characterized by elevated levels of reactive oxygen species (ROS) and the resultant high OS, a hallmark of malignant tumors 1. Various risk factors contributing to OS are intricately linked to the pathogenesis and severity of BC 2,3. Cancer formation is a complex, multi-stage, and gradual evolutionary process primarily driven by the accumulation of genetic mutations and dysregulation of cell division 4. In this deep-rooted pathological transition mechanism, intracellular OS plays a crucial catalytic role by promoting cancer development through progressive disruption of genomic stability and integrity and interaction with key cellular macromolecules, including DNA, RNA, and proteins 5.

However, the relationship between OS and cancer is not non-linear. OS, particularly elevated ROS homeostasis, is closely linked to an increased risk of cancer development 6, playing a role at every stage from initiation to progression 7. Studies have shown a significant positive correlation between OS and postmenopausal BC risk 8-10. In contrast, OS signaling also exerts pivotal influences on processes such as apoptosis and ferroptosis 7,11, which may have preventive effects against cancer in premenopausal women. Two prospective studies focusing on premenopausal women have demonstrated that higher OS levels were linked to a reduced risk of BC 12,13.

Given the dual role of OS, it is critical that BC researchers use a multi-omics approach to explore its impact. The OS-associated genome includes more than a thousand nuclear genes whose genetic susceptibility can profoundly influence OS status. However, only a limited number of studies have examined the pathogenic role of OS-associated genes in BC aetiology. Understanding the pathogenesis of these genes could help identify causative factors and potential redox-related therapeutic targets in BC 14. Rigorous and comprehensive analyses of OS-related genes in the context of BC are essential to determine whether OS is a cause or a consequence of BC. Such analyses are particularly important in light of current challenges to BC treatment, including the development of drug resistance, which may be influenced by OS-related mechanisms 15. Exploring these mechanisms could reveal new therapeutic strategies that may provide more effective treatment options and improve patient prognosis.

Despite growing evidence of an association between OS-related genes and BC, comprehensive and systematic investigations into their causal relationship remain scarce. Genome-wide association studies (GWAS) have been employed to identify genomic loci associated with BC risk 16-18. However, due to the complex linkage disequilibrium (LD) structure of the genome, the top-associated variants identified in GWAS may not be causal 19,20. Furthermore, these genetic variants can influence DNA methylation, gene expression, alternative splicing, and protein abundance. Integrated multi-omics approaches have emerged as valuable tools in the post-GWAS era for identifying key regulatory elements and exploring potential therapeutic targets in BC. Advances in GWAS and molecular quantitative trait loci (QTL) datasets now enable a detailed investigation into the causal relationship between the regulation of OS-related genes and BC, with a focus on methylation, gene expression, alternative splicing, and protein abundance. This study employs a range of post-GWAS techniques to assess the association between OS gene methylation, expression, alternative splicing, and protein abundance with BC risk.

## Methods

### Study Design

**Figure 1:**
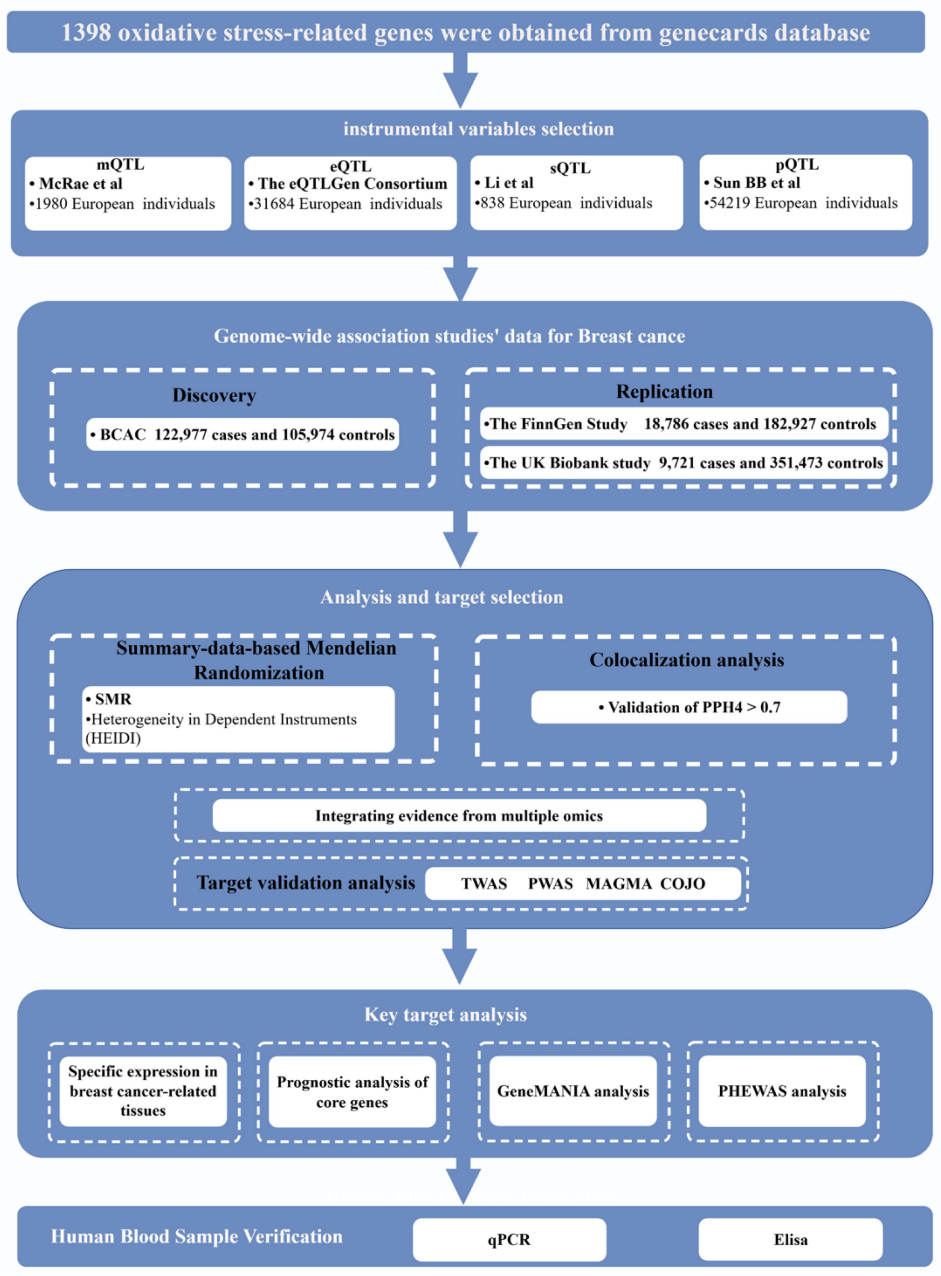
Study design. SMR, summary-based Mendelian randomization; QTL, quantitative trait loci; BC, Breast Cancer; PPH4, posterior probability of H4.

Figure 1 illustrates the comprehensive design of the study. This analysis utilized publicly available datasets, including the BC Association Consortium (BCAC) 17, the UK Biobanking Study 21, the Finnish Genetics Study 22, and other large-scale GWAS (Table S1). Instrumental variables for OS genes were derived from methylation levels, alternative splicing RNA, gene expression, and protein abundance levels. Subsequently, summary-based Mendelian randomization (SMR) analyses were conducted for BC across these biological levels, complemented by transcriptome-wide association studies (TWAS), proteome-wide association studies (PWAS), and multi-marker analysis of genomic annotation (MAGMA) analyses for multiple validation. Furthermore, co-localization analyses were performed to reinforce the plausibility of the causal relationship. The BCAC dataset was the primary discovery dataset, while replication analyses used datasets from the UK Biobank and the Finnish Genetics Study. Specifically, these datasets were used to study gene methylation, alternative splicing RNA, gene expression, and protein abundance levels. By integrating the results of these four different levels of analysis, we identified causal candidate genes without sample overlap between exposed and outcome populations.

### Data sources for methylation, alternative splicing RNA, gene expression, and protein quantitative trait loci

Multi-omics integration enables the identification of potential molecular networks associated with OS. QTL can be used to elucidate the relationships between single nucleotide polymorphisms (SNPs) and DNA methylation, alternative splicing RNA, gene expression, and protein abundance. The methylation QTL (mQTL) data were obtained from Wu's study 20 on SNP-CpG associations in blood samples from individuals of European ancestry. The blood expression QTL (eQTL) dataset was sourced from the eQTLGen consortium 23, which encompasses 31,684 individuals. A synopsis of the genetic statistics associated with circulating protein levels was extracted from the protein QTL (pQTL) study by Sun et al. 24, which included 54,219 participants. Subsequently, the tissue-specific expression of the target genes was assessed using whole blood splicing QTL (sQTL) data retrieved from the Genotypic Tissue Expression (GTEx) Portal (https://gtexportal.org/home/). Additionally, tissue-specific expression was examined. The QTL data demonstrated a potential causal effect between the target genes and BC 25. The GTEx v8 dataset comprises 838 donors and 17,382 samples from 52 tissues and two cell lines. Analysis of BC-associated tissues included eQTL data for thyroid, breast tissue, muscle tissue, and subcutaneous fat.

Gene expression data from whole blood, utilized for TWAS analysis, were sourced from the FUSION website (http://gusevlab.org/projects/fusion/) from Alexander Gusev's study 26, while the enriched gene expression data from four BC-related tissues were obtained from the GTEx V8 dataset. The PWAS protein weights used for the analysis were obtained from Zhang's study 27.

OS genes were identified using GeneCards, and a list of 1,398 OS-related genes with correlation scores of ≥7 was downloaded from the GeneCards database (https://www.genecards.org) 28. This list was then used to identify OS genes in the QTL dataset.

### BC GWAS summary data source

BC data were obtained from the BCAC, the FinnGen study, and the UK Biobank study. All participants in the BCAC study were of European ancestry, comprising 122,977 patients with BC and 105,974 controls. Furthermore, summary-level data on genetic associations with BC were obtained from the FinnGen study's publicly available R10 dataset, which included 18,786 patients with BC and 182,927 controls. Furthermore, BC-related summary data were sourced from the UK Biobank database, comprising 9,721 cases and 351,473 controls. The discovery phase of the study employed the BCAC dataset, while the replication phase utilized data from the UK Biobank and Finnish studies. Notably, no sample overlap occurred between these three datasets.

### SMR analysis

To study the link between gene regulation (methylation, expression, alternative splicing, and protein abundance) and BC risk, we used SMR. This method uses genetic variants as instruments to estimate causal effects using summary data. Compared to traditional MR, SMR has higher statistical power when using two large, independent samples 29. We selected cis-QTLs within a ±1000 kb window around each gene, with a P-value threshold of 5.0 × 10⁻⁸. SNPs with significant allele frequency differences across datasets were excluded. The HEIDI test helped differentiate pleiotropy from linkage, and variants with P-HEIDI < 0.01 were excluded. A nalyses were performed using SMR v1.3.1, and P-values were adjusted with the Benjamini-Hochberg method to control the false discovery rate at 0.05. Co-localization analysis was then done for significant results.

### Co-localization analysis

To further validate our results, we performed co-localization analysis using the coloc R package. This analysis helps identify shared causal variants between BC and the identified OS-related mQTLs, eQTLs, sQTLs, or pQTLs 30. According to published articles, the co-localization region windows for pQTL-GWAS 31, eQTL-GWAS 31, sQTL-GWAS 32, and mQTL-GWAS 33 were ±1,000 kb, ±1,000 kb, ±1,000 kb, and ±500 kb, respectively. We used a Bayesian approach to assess support for five hypotheses: no association with either trait (H0), association with trait 1 only (H1), association with trait 2 only (H2), association with both traits but with different causal variants (H3), and association with both traits with similar causal variants (H4).For each locus, we set the prior probabilities for an SNP being associated only with trait 1 (p1), only with trait 2 (p2), or with both traits (p12) to 1 × 10⁻⁴, 1 × 10⁻⁴, and 1 × 10⁻⁵, respectively. A posterior probability of a shared causal variant (PP.H4) ≥ 0.70 was considered strong evidence for co-localization, with a corresponding FDR < 5%. This strengthens the evidence for a causal relationship 34.

### Proteome/transcriptome-wide association studies with fusion

FUSION software 26 was used to establish associations between functional and GWAS phenotypes and to perform PWAS and TWAS analyses. It was also used to validate the association between protein/gene expression levels and BC susceptibility. The main outputs of FUSION are Z-scores and p-values, where Z-scores quantify the strength and direction of the association between plasma proteins and BC, and p-values elucidate the statistical significance of the association. To further validate our findings at the transcriptome level, TWAS was used on thyroid, breast, muscle, and subcutaneous adipose tissues.

### Conditional and joint analysis

FUSION helps identify multiple related traits within a locus and determine which of these are conditionally independent. Therefore, we performed conditional and joint (COJO) analyses (a post-processing module in FUSION) to identify independent genetic signatures 26. COJO analyses provide a comprehensive understanding of the genetic architecture of trait variation by interpreting LD between markers 35. Genes with independent associations were termed jointly significant, while those without independent associations were termed marginally significant.

### Gene-level analysis

For genetic analyses, we used MAGMA software (version 1.08) with default parameters. This tool aggregates SNP-level association statistics into gene scores, enabling quantification of each gene's association with the phenotype 36,37. Further details on parameter settings and the methodological basis are available in the original MAGMA documentation 38.

### Integrating results at the multi-omics level of evidence

For a comprehensive understanding of how the regulation of OS-related genes correlates with BC at different levels, we integrated results from four different gene regulatory levels. Given that proteins are the final expression products of genes and establishing causality at the protein level is the most basic requirement, we integrated the resulting genes that were correlated at multiple levels to explore further the potential regulation between gene methylation, expression, alternative splicing, and protein abundance. We performed MR analyses of OS-associated gene methylation, gene expression, alternative splicing, and protein abundance between the causal relationships. Moreover, we conducted co-localization analyses of the identified associations to exclude the effects of LD.

### Survival analysis

The Kaplan-Meier plotter database (http://kmplot.com) is a survival analysis tool that integrates data from public databases, including GEO (Gene Expression Omnibus), EGA (European Genome-phenome Archive), TCGA (The Cancer Genome Atlas), and other public databases for breast cancer (BC) patients. This tool can be used to assess disease progression 39.

### Quantitative Reverse Transcription-Polymerase Chain Reaction (RT-PCR)

A total of 16 subjects were recruited for this study, including 8 newly diagnosed, untreated breast cancer patients from Weifang Hospital of Traditional Chinese Medicine and 8 healthy volunteers. Prior to the initiation of the study, all patients provided written informed consent. All breast cancer patients were confirmed to be primary, untreated cases, ensuring that no prior treatments (such as chemotherapy or radiotherapy) could confound the expression levels of PARK7 (also known as DJ-1). Samples were collected from each participant prior to initial treatment. Total RNA was isolated from whole blood using the Whole Blood RNA Isolation Kit (Simgen), and the isolated total RNA was reverse transcribed using the cDNA Strand Synthesis Kit (Accurate Biotechnology). RT-PCR was performed using the BIO-RAD CFX96 Sequence Detection System. Human PBX samples were analysed by using the SYBR Green PCR Master Mix (Accurate Biotechnology) for human PARK7. Relative levels of gene expression were determined by the comparative threshold cycling method as described by the manufacturer.

### ELISA assay for PARK7

PARK7 levels in human blood were assessed by enzyme-linked immunosorbent assay (ELISA) using a commercially available Human PARK7 ELISA Kit (#CSB-E12024h, CHUANGABIO, Wuhan, China) according to the manufacturer's protocol.

### GeneMANIA analysis

The GeneMANIA platform 40 (https://genemania.org/) integrates multiple genetic interactions, pathways, and co-expression datasets of target genes along with other gene function relationships to enhance understanding of the potential biological functions of these targets 41.

### Phenome-wide association analysis

Using the ExPheWeb platform (https://exphewas.statgen.org/v1/), phenome-wide association studies (PheWAS) were performed to assess the pleiotropic effects and potential adverse effects of potential therapeutic targets 42. The ExPheWas database contains 26,616 genes measured in up to 413,133 individuals from UK biobanks with 1,746 phenotypes based on sex-stratified and sex-combined gene association results. This comprehensive PheWAS analysis offers insights into understanding complex genetic traits and assessing the safety and efficacy of drug targets.

## Results

### Identification of correlations at the methylation level

**Figure 2:**
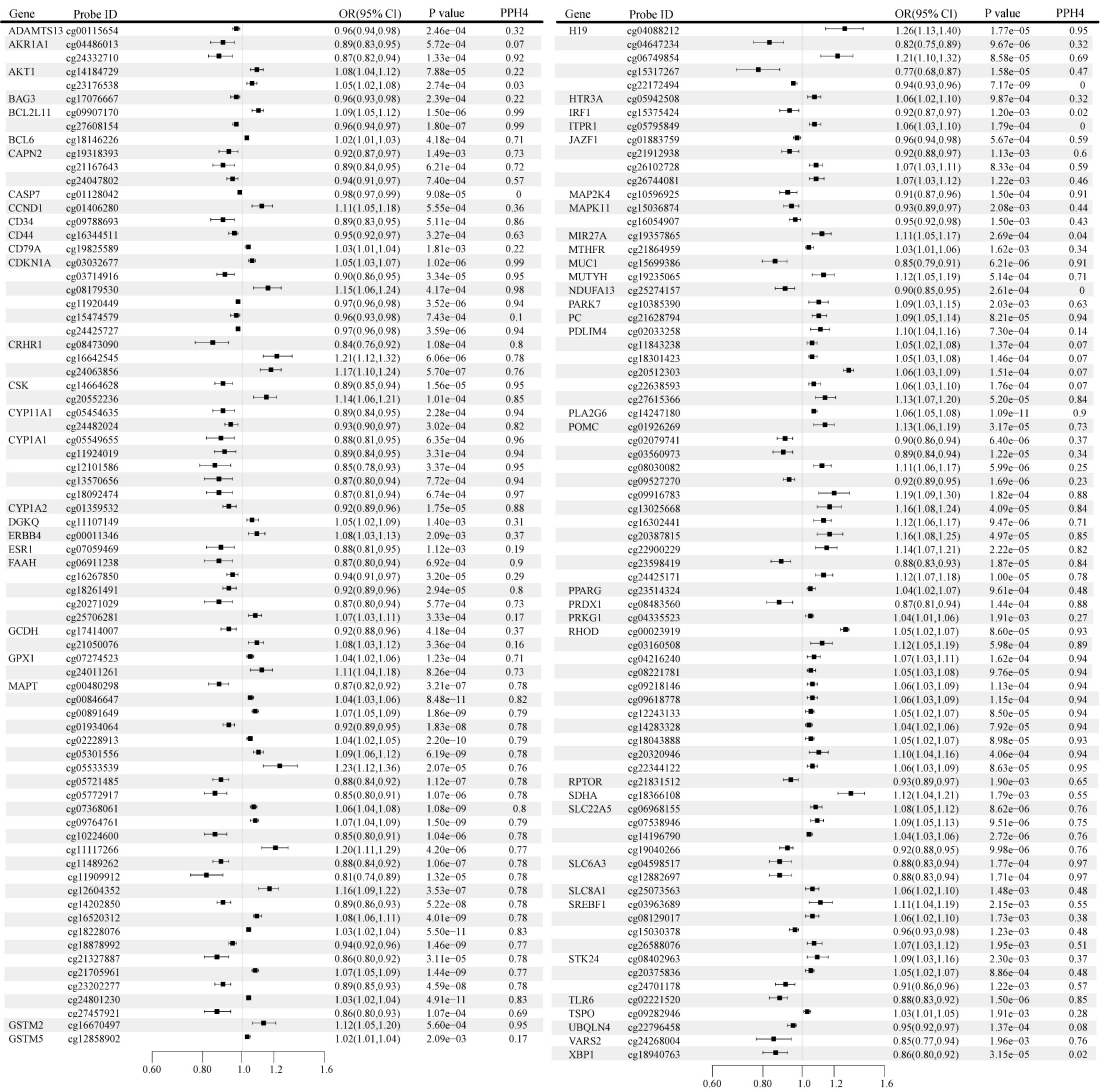
Genetically predicted association of oxidative stress gene methylation with breast cancer. OR, odds ratio; CI, confidence interval.

The causal effects of OS gene methylation on BC risk are shown in Figure 2. A total of 613 CpG sites near 250 unique genes passed the marginal significance test (P < 0.05) after excluding associations with P-HEIDI < 0.01 (Table S2). Following multiple testing corrections, we identified 151 CpG sites near 60 unique genes, including 71 sites near 29 unique genes that demonstrated strong evidence of co-localization (PH4 > 0.70) (Figure 2). Notably, the direction of effect estimates was not always consistent for different CpG sites within the same gene. For instance, the gene located at cg11909912 predicted that a one-fold increase in microtubule-associated protein tau (MAPT) methylation was associated with a reduced risk of BC (odds ratio [OR]: 0.81, 95% confidence interval [CI]: 0.74-0.89), whereas the gene located at cg11117266 predicted that a one-fold increase in MAPT methylation was associated with an increased risk of BC (OR: 1.20, 95% CI: 1.11-1.29).

The CpG loci of interest identified included CCND1 (cg01406280), SLC22A5 (cg06968155, cg07538946, cg14196790, cg19040266), MAP2K4 (cg10596925), DGKQ (cg11107149), FAAH (cg16267850), also replicated in FinnGen & UK Biobank, CYP1A2 (cg01359532), TLR6 (cg02221520), H19 (cg04088212), CYP1A1 (cg05549655, cg11924019, cg12101586. cg13570656, cg18092474), IRF1 (cg15375424), SDHA (cg18366108) replication confirmed in UK Biobank, FAAH (cg06911238, cg18261491, cg20271029), SLC6A3 (cg12882697), MAPK11 (cg15036874, cg16054907), BAG3 (cg17076667), UBQLN4 (cg22796458) replicates were obtained in FinnGen (Table S3).

### Identification of correlations at the transcriptome level

**Figure 3:**
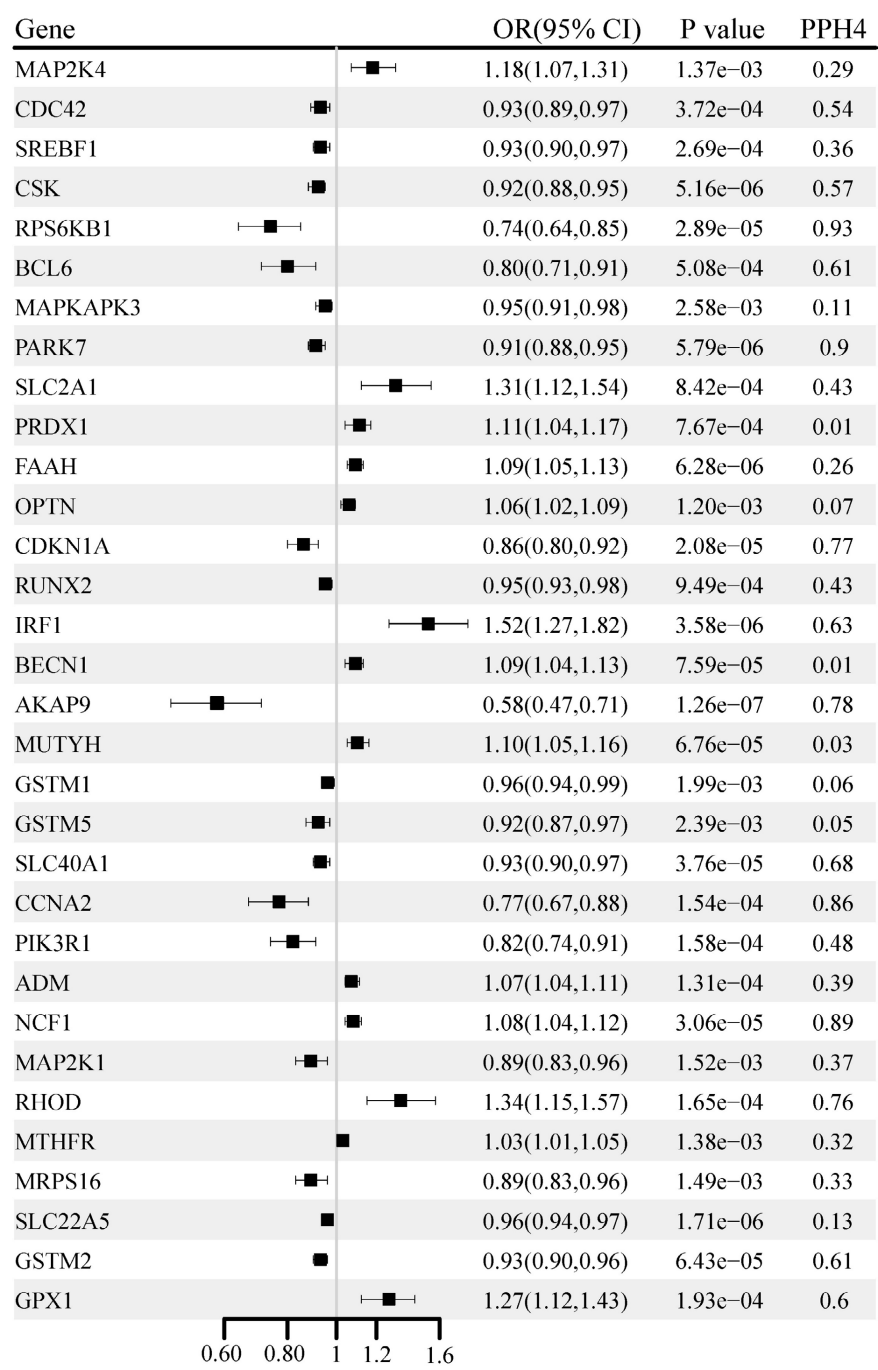
Genetically predicted association of oxidative stress gene expression with breast cancer.

The causal associations of OS gene expression with BC are shown in Figure 3. A total of 126 genes were significantly associated with BC (P < 0.05) (Table S4). After multiple testing corrections and co-localization analysis, higher levels of gene-predicted expression, including NCF1 (OR: 1.08, 95% CI: 1.04-1.12; PPH4 = 0.89), RHOD (OR: 1.34, 95% CI: 1.15-1.57; PPH4 = 0.76) were positively associated with BC risk. In contrast, the genetically predicted RPS6KB1 (OR: 0.74, 95% CI: 0.64-0.85; PPH4 = 0.93), PARK7 (OR: 0.91, 95% CI: 0.88-0.95; PPH4 = 0.90), CDKN1A (OR: 0.86, 95% CI: 0.80-0.92; PPH4 = 0.77), AKAP9 (OR: 0.58, 95% CI: 0.47-0.71; PPH4 = 0.78), and CCNA2 (OR: 0. 77, 95% CI: 0.67-0.88; PPH4 = 0.86) were negatively correlated with BC risk. The correlation for PARK7 and CDKN1A was replicated in the UK Biobank cohort, and the correlations for RPS6KB1, AKAP9, and CCNA2 were replicated in the FinnGen cohort (Table S5).

### Identification of correlations at the alternative splicing level

**Figure 4:**
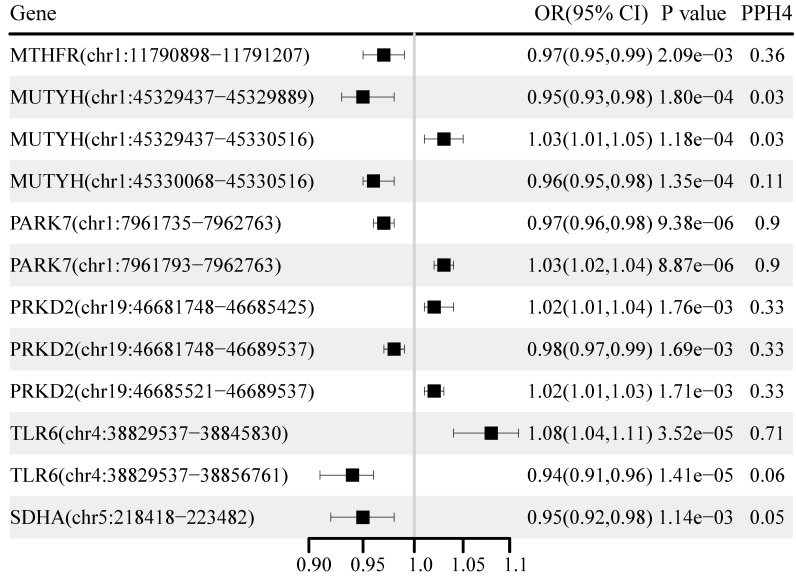
Genetically predicted association of alternative splicing of oxidative stress gene with breast cancer.

The causal effects of the OS gene alternative splicing RNA on BC and its subtypes are shown in Figure 4. A total of 46 loci near the 24 unique genes passed the significance test (P < 0.05) after excluding associations with a P-HEIDI of < 0.01 (Table S6). After correction for multiple testing, we found 12 loci near six unique genes. Among them, PARK7 chr1:7961735:7962763 (OR: 0.97, 95% CI: 0.96-0.98), PARK7 chr1:7961793:796276 (OR: 1.03, 95% CI: 1.02-1.04), and TLR6 chr4:38829537:38845830 (OR: 1.08, 95% CI: 1.04-1.11) showed strong evidence of co-localization (PH4 > 0.70). TLR6 chr4:38829537:38845830 was replicated in the UK Biobank and FinnGen cohorts, while both sQTL loci for PARK7 were replicated in the UK Biobank cohort (Table S7).

### Identification of associations at the proteomic level

**Figure 5:**
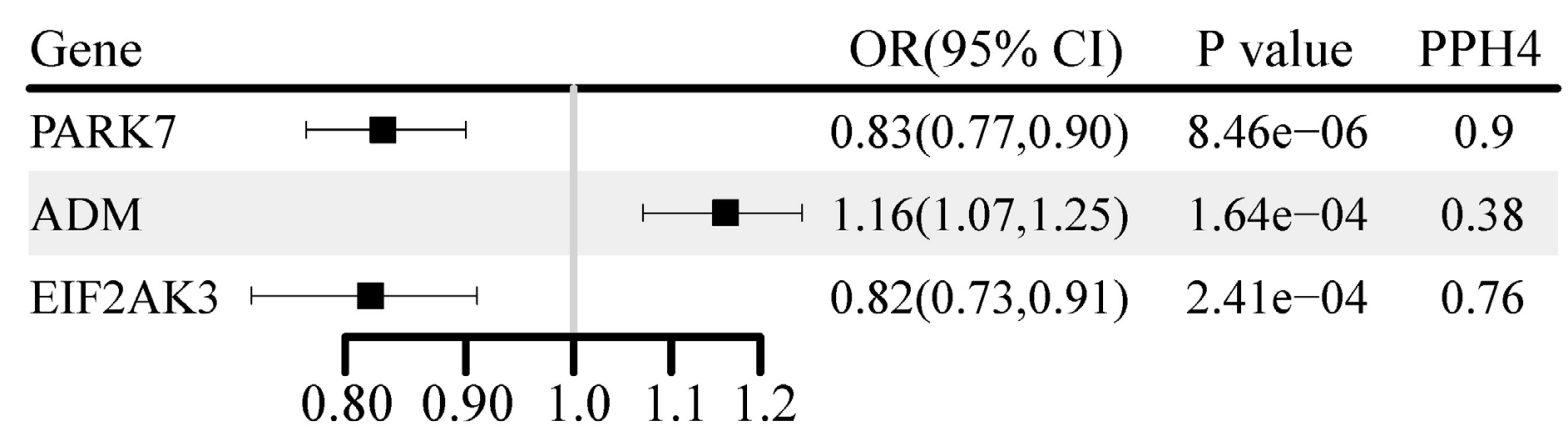
Genetically predicted association of oxidative stress gene encoded protein with breast cancer.

Fifty-one OS proteins were associated with BC risk (P < 0.05) (Table S8). Three were corrected for multiple testing, and genetically predicted higher levels of PARK7 and EIF2AK3 were negatively associated with BC risk (Figure 5). Genetically predicted higher levels of adrenomedullin (ADM) were positively associated with BC risk. PARK7 (OR: 0.83, 95% CI: 0.77-0.90) and EIF2AK3 (OR: 0.82, 95% CI: 0.73-0.91) demonstrated a significant increase in BC risk with evidence of high co-localization (PH4 ≥ 0.7). This association was replicated in the UK Biobank cohort for PARK7 and in the FinnGen cohort for EIF2AK3 (Table S9).

**Figure 6:**
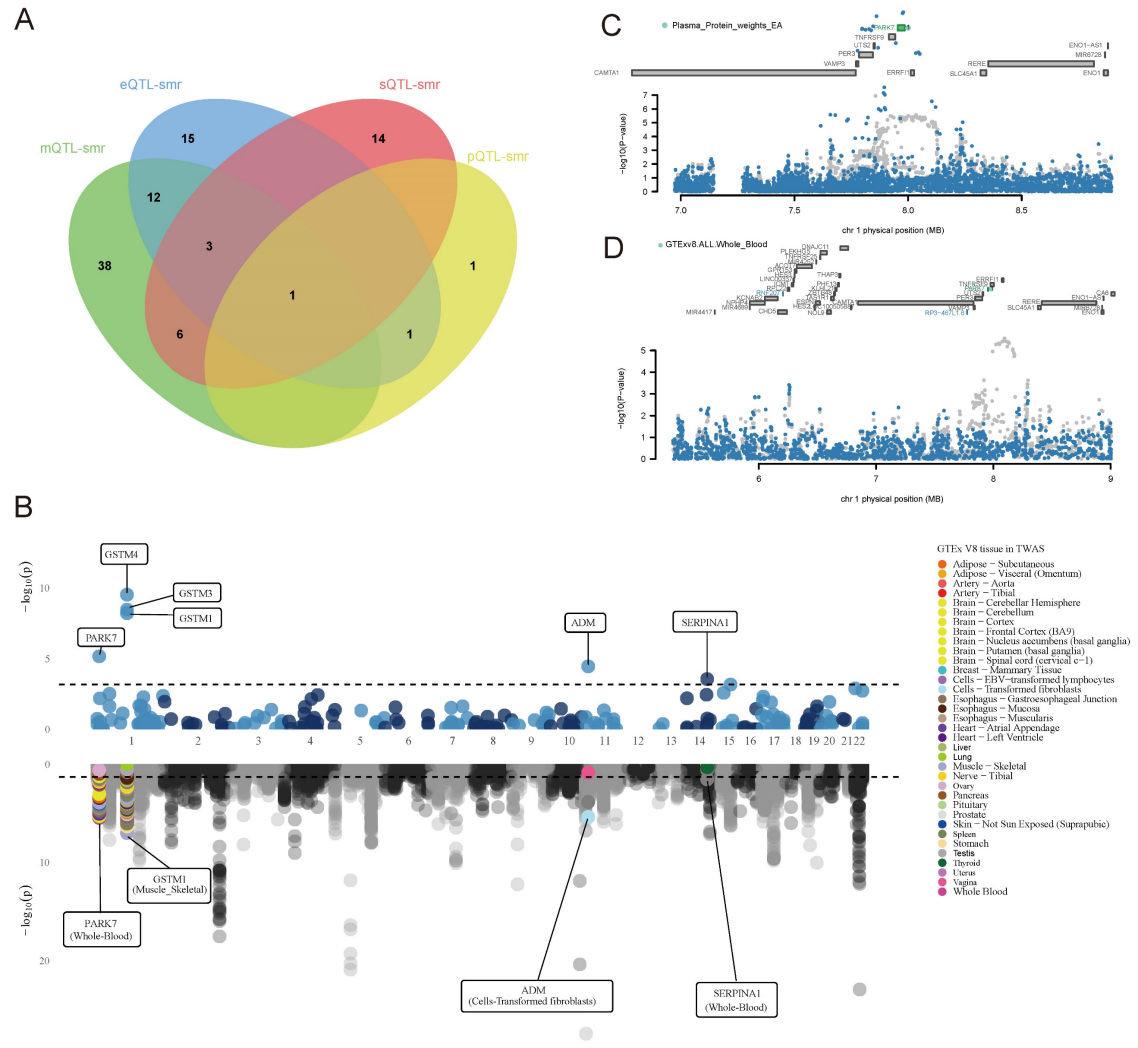
Core gene identification and validation. (A) Core gene identification. (B) Miami plot for PWAS (upper) and TWAS (lower) of breast cancer. (C) Regional association of TWAS hits. The top panel highlights all genes in the region. The marginally associated TWAS genes are shown in blue, and the jointly significant genes are shown in green. The bottom panel shows a regional Manhattan plot of GWAS data before (grey) and after (blue) conditioning on the predicted expression of the green genes.

We superimposed the results of the above multi-omics analyses and identified a core gene, PARK7, that was associated with BC risk at multiple dimensional levels (Figure 6A).

### Target validation analysis

In FUSION PWAS analysis of blood proteins, seven OS-associated proteins with FDR < 0.05 were identified (Table S10), with PARK7 (PH4 = 0.977) showing evidence of high co-localization. Tissue-specific FUSION TWAS analysis identified 177 OS-related genes with FDR < 0.05 in at least one tissue, with PARK7 being significantly associated with BC in multiple tissues (Figure 6B and Table S11). PARK7 was further validated as a significant OS gene associated with BC risk (FDR < 0.05) in a MAGMA gene-based analysis (Table S12).

We performed COJO analyses for PARK7 in whole blood gene expression and protein to eliminate LD-induced false positives and assess the independence of PARK7 expression in whole blood protein analyses (Figure 6C). In whole blood gene expression, the TWAS signals of RNF207 and RP3-467L1.6 showed a significant decrease when the analysis was conditioned on the predicted expression of PARK7 (Figure 6D).

### Integrating evidence from multiple omics

After integrating multi-omics evidence, PARK7 correlated with BC risk in several biological processes. Gene expression of PARK7 positively correlated with its corresponding protein levels. Methylation at cg10385390 in PARK7 was associated with lower PARK7 gene expression, which is consistent with the positive effect of cg10385390 methylation on BC risk. Similarly, methylation at cg11518359 in PARK7 was associated with higher expression of the PARK7 gene, which is also consistent with the negative effect of cg11518359 methylation on BC risk. PARK7 (chr1:7961793-7962763) increases BC risk by reducing PARK7 expression, whereas PARK7 (chr1:7961735-7962763) decreases BC risk by upregulating PARK7 expression. Co-localization analyses provided strong evidence (PPH4 > 0.70) for mQTL-eQTL, mQTL-pQTL, sQTL-pQTL, and eQTL-pQTL relationships involving the PARK7 gene (Table S13).

### Specific expression of PARK7 in BC-related tissues and prognostic analysis

**Figure 7:**
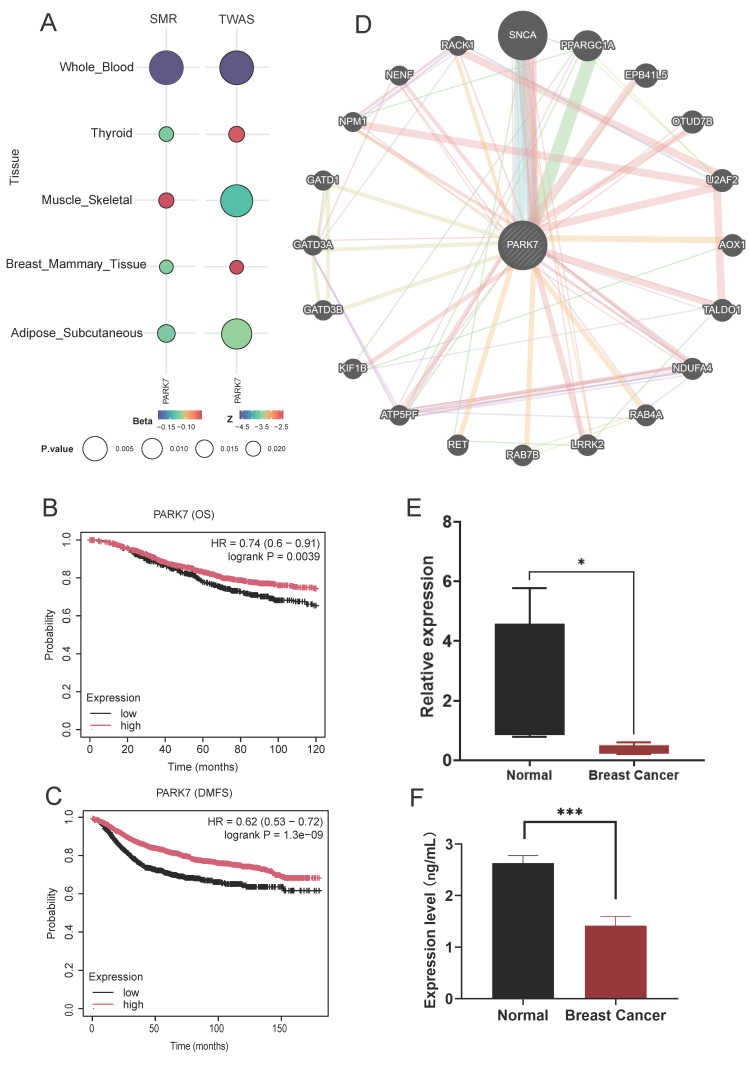
(A) Correlation of PARK7 with breast cancer in breast cancer-associated tissues. (B) Effect of PARK7 Expression on Overall Survival in Breast Cancer Patients. (C) Effect of PARK7 Expression on DMFS in Breast Cancer Patients. (D) GeneMANIA analysis of PARK7 (E) qPCR results showed that the expression levels of PARK7. (F) Elisa results showing protein levels of PARK7.

We further explored the causal relationship between the identified core genes and the tissues associated with BC, with genetically predicted PARK7 expression levels inversely correlating with BC risk in subcutaneous fat, breast, musculoskeletal, and thyroid tissues. In TWAS validation, PARK7 also significantly correlated with BC in the above tissues and blood proteins (Figure 7A). Meanwhile, in patients with BC, high PARK7 expression was significantly associated with longer overall survival (Figure 7B) and distant metastasis-free survival (Figure 7C).

### GeneMANIA analysis

Figure 7D demonstrates the network of potentially interacting genes constructed with PARK7 as the core. The top functional pathways enriched in the PARK7-associated gene network were regulation of neuron death, negative regulation of response to ROS, negative regulation of OS, regulation of hydrogen peroxide-induced cell death, cellular response to hydrogen peroxide, and regulation of cellular response to hydrogen peroxide (Table S14).

### Validation of gene and protein expression of PARK7 by qPCR and ELISA

The expression of the PARK7 gene was validated in blood samples by qPCR. The results demonstrated that the mRNA levels of PARK7 in plasma from breast cancer patients were significantly lower than those in the healthy control group (Fig. 7E). Serum PARK7 protein levels were further analyzed using enzyme-linked immunosorbent assay (ELISA) in both breast cancer patients and healthy controls. We observed a marked reduction in PARK7 protein levels in breast cancer patients (Fig. 7F).

### PheWAS

PheWAS was performed using the ExPheWas platform, focusing on PARK7 as an exposure factor to explore its potential side effects. After MR analysis of 1,505 diseases or traits and applying Bonferroni correction, only mean platelet volume was significantly associated with PARK7, while none of the other traits showed significant associations at the gene level (Table S15). This suggests that both the potential side effects of drug action on PARK7 targets and the likelihood of horizontal pleiotropy from this gene are low, confirming the reliability of our findings. Furthermore, when PARK7 was used as a therapeutic target, the risk of inducing side effects or unintended horizontal pleiotropic effects was relatively low, further strengthening the validity and applicability of the results of this study.

## Discussion

To our knowledge, this study is the first to systematically elucidate the potential causal mechanisms between OS-related genes and BC using an integrative multi-omics strategy. Based on blood multi-omics data, we not only revealed the causal effects of OS on BC characterized by genetic susceptibility but also identified the key regulatory gene PARK7, which exhibited robust causal associations with BC risk across multiple molecular dimensions, including methylation, gene expression, alternative splicing, and protein abundance. Moreover, we validated the gene expression and protein abundance of PARK7 in an independent clinical cohort, with results consistent with the multi-omics predictions, thereby experimentally supporting the reliability of PARK7 as a risk factor for BC.

Recently, blood biomarkers have emerged as valuable tools for diagnosing, monitoring, and assessing the prognosis of BC. Blood tissues offer a crucial window into the complex etiology of BC, including the genetic effects of gene expression. Recognizing proteins as the direct executors of gene functions, our analysis established a causal chain of evidence at the protein level, representing an indispensable link for research. Through SMR analysis, we assessed methylation, alternative splicing, gene expression, and protein abundance in whole blood samples. We identified three putative causal associations with BC susceptibility involving PARK7, ADM, and EIF2AK3 genes. Among these, PARK7 showed a significant causal association with BC at multiple levels of analysis. To further validate this finding, we conducted extensive post-GWAS analyses to consolidate the original findings and investigate the tissue-specific expression of PARK7 and its therapeutic potential.

PARK 7, also known as DJ-1 protein, is widely expressed in cells and plays a critical role in regulating cellular responses to OS, protecting mitochondrial function, maintaining cellular redox homeostasis, and inhibiting apoptosis 43. The present study demonstrated that decreased levels of circulating PARK7 protein abundance were associated with an increased risk of BC. A retrospective study by Tsuchiya reported reduced DJ-1 protein expression in BC invasive ductal carcinoma (IDC) tissue compared to adjacent non-cancerous epithelial tissue. Moreover, in patients with IDC, lower DJ-1 protein expression was significantly associated with shorter disease-free survival (P = 0.015) and overall survival (P = 0.020) 44, which is consistent with the results of our analysis. However, an observational study noted that DJ-1 was upregulated in hormone receptor-positive BC and associated with poor prognosis 45. These conflicting findings suggest a more complex relationship between PARK7 and BC, which requires further investigation. Evidence for an association between PARK7 and BC from observational, epidemiological, and experimental studies is scarce, and these discrepancies may be due to the limitations of adjusting for confounders and reverse causality in traditional epidemiological studies. Therefore, the relationship between PARK7 and BC risk remains uncertain.

Given these considerations, the present study is of significant importance. Existing research has predominantly focused on individual molecular levels, lacking an integrative analysis of multi-omics regulatory networks, which has hindered a comprehensive understanding of the complex associations between PARK7 and breast cancer (BC). By integrating mQTL, eQTL, sQTL, and pQTL data, our study, for the first time, elucidates the bidirectional regulatory roles of PARK7 methylation sites (cg10385390 and cg11518359) and splicing variants (chr1:7961793-7962763 and chr1:7961735-7962763). These findings provide a molecular basis for resolving the directional contradictions in PARK7 expression observed across different studies and potentially address the controversies regarding the association between PARK7 and BC. Traditional epidemiological studies often struggle to fully control for confounding factors or rule out reverse causality. In contrast, our study employs Mendelian randomization (MR) and colocalization analyses, leveraging genetic instrumental variables to infer causal associations and significantly reduce the risk of bias. This approach offers more reliable evidence for clarifying the causal relationship between PARK7 and BC risk. Moreover, through validation in an independent clinical cohort using blood samples, we confirmed the systemic downregulation of PARK7 mRNA and protein levels in BC patients, providing direct evidence for the tissue consistency of causal associations and further corroborating the reliability of our multi-omics analysis results. The dynamic methylation and regulatable splicing variants of PARK7 suggest its potential as a subtype-specific therapeutic target, and the discoveries made in our study offer potential directions for developing personalized treatment strategies targeting specific BC subgroups, thereby advancing the application of precision medicine in the field of breast cancer.

Additionally, tissue-specific analyses revealed consistent expression patterns of PARK7 across thyroid, breast, muscle, and subcutaneous adipose tissues compared to blood, suggesting its potential to perform similar biological functions across different tissues and supporting its potential value as a breast cancer biomarker. However, further experimental and observational studies are needed to clarify the directionality of the association between PARK7 and BC.

A key strength of our study is the utilisation of SMR and co-localisation, which collectively leverage genetic variation to estimate the causal effects of oxidative stress gene methylation, variable shear, expression and protein abundance. Employing a range of post-GWAS analyses for validation, in conjunction with independent biological validation of the population cohort, has further enhanced the reliability of our results. Specifically, we examined PARK7 mRNA expression levels and protein abundance in human blood samples by RT-qPCR and ELISA. In addition, we integrated results from multi-omics level evidence that strengthened the causal relationship between oxidative stress-related genes and BC risk. MR designs complementarily minimise bias from confounding and reverse causation, thereby improving causal inference, and co-localisation methods have been shown to be a powerful tool for eliminating potential bias due to linkage disequilibrium. Furthermore, GWAS with a large sample size increased the statistical power of our study, and the consistency of our results across multiple datasets provides additional support for our findings.

It is imperative to acknowledge the limitations of the present study. The current analyses are constrained by the availability of data. The multi-omics analyses have been conducted on whole blood tissues and have only been expanded to encompass gene expression analysis for breast cancer-related tissues. It has not been feasible to estimate associated methylation, alternative splicing, and protein levels in other tissues. Evaluating the role of proteins and other levels in breast cancer in other tissues may offer additional insights into the pathogenesis of breast cancer, particularly in breast tissue. Additionally, the findings of this study are primarily based on multi-omics data from European populations, who constitute only a portion of the global population. Although the large sample size ensures statistical power, genetic and epigenetic regulatory differences across ethnicities may limit the generalizability of our conclusions. With the increasing accessibility of multi-omics sequencing technologies, future studies are expected to validate the cross-ethnic consistency of the PARK7-breast cancer association in more diverse populations.

## Conclusions

This study provides novel insights into the causal role of oxidative stress (OS) in breast cancer (BC) development through an integrative multi-omics Mendelian randomization (MR) approach, identifying PARK7 as a key driver in BC pathogenesis. Our findings advance the understanding of the molecular mechanisms underlying BC and highlight PARK7's potential as a therapeutic target and biomarker. Future research should focus on further validating these mechanisms and exploring their clinical value in BC patients. With the advancement of high-throughput omics technologies, integrating multi-omics data to reveal BC's complex pathophysiology will be crucial, offering promise for identifying additional therapeutic targets and improving personalized treatment strategies.

## Supplementary Material

Supplementary tables.
